# Interleukin-1 Links Autoimmune and Autoinflammatory Pathophysiology in Mixed-Pattern Psoriasis

**DOI:** 10.1155/2021/2503378

**Published:** 2021-10-16

**Authors:** Rodolfo Kölliker Frers, Matilde Otero-Losada, Tamara Kobiec, María Inés Herrera, Lucas Udovin, Carlos F. Kusnier, Francisco Capani

**Affiliations:** ^1^Centro de Altos Estudios en Ciencias Humanas y de la Salud, Universidad Abierta Interamericana, Consejo Nacional de Investigaciones Científicas y Técnicas, CAECIHS-UAI.CONICET, Argentina; ^2^Departamento de Reumatología, Hospital J. M. Ramos Mejía, Buenos Aires, CABA, Argentina; ^3^Centro de Investigaciones en Psicología y Psicopedagogía (CIPP), Facultad de Psicología y Psicopedagogía, Pontificia Universidad Católica Argentina (UCA), Buenos Aires, Argentina; ^4^Universidad JF Kennedy, Buenos Aires, Argentina; ^5^Universidad Autónoma de Chile, Santiago, Chile

## Abstract

Autoinflammatory and autoimmune diseases are characterized by an oversensitive immune system with loss of the physiological endogenous regulation, involving multifactorial self-reactive pathological mechanisms of mono- or polygenic nature. Failure in regulatory mechanisms triggers a complex network of dynamic relationships between innate and adaptive immunity, leading to coexistent autoinflammatory and autoimmune processes. Sustained exposure to a trigger or a genetic alteration at the level of the receptors of the natural immune system may lead to abnormal activation of the innate immune system, adaptive system activation, loss of self-tolerance, and systemic inflammation. The IL-1 family members critically activate and regulate innate and adaptive immune responses' diversity and plasticity in autoimmune and/or autoinflammatory conditions. The IL-23/IL-17 axis is key in the communication between innate immunity (IL-23-producing myeloid cells) and adaptive immunity (Th17- and IL-17-expressing CD8+ T cells). In psoriasis, these cytokines are decisive to the different clinical presentations, whether as plaque psoriasis (psoriasis vulgaris), generalized pustular psoriasis (pustular psoriasis), or mixed forms. These forms reflect a gradient between autoimmune pathophysiology with predominant adaptive immune response and autoinflammatory pathophysiology with predominant innate immune response.

## 1. Introduction

**Figure 1:**
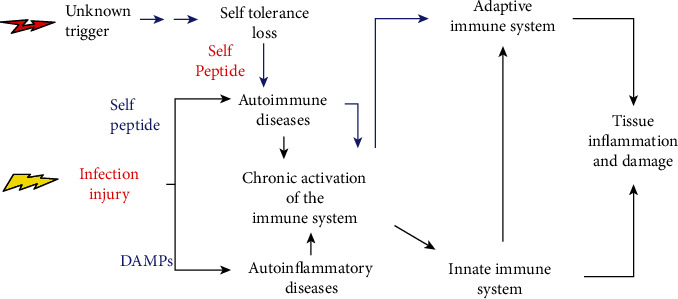
Simplified representation of the relative contribution of autoimmune, autoinflammatory, and mixed-pattern forms to tissue damage. DAMPs: damage-associated molecular patterns.

Autoinflammatory and autoimmune diseases are characterized by immune system hyperactivity, typically featuring an against-self pathological process. They are systemic diseases and mono- or polygenic. The innate immune system directly causes tissue inflammation in autoinflammatory diseases. An adaptive immune dysregulation—against self—is found in autoimmune diseases. Both combined are present in mixed autoinflammatory-autoimmune pattern diseases (Figure 1).

The former characterization of autoinflammatory diseases, the more recent mixed form presentation (autoinflammatory-autoimmune), and the changing contribution of underlying autoinflammatory processes to autoimmunity pathways further complicated understanding the pathophysiological processes [1].

The immune system responds to independent pathways, either exogenous (bacteria) or endogenous (injured tissue), yet with crosscommunication.

The clinical diversity of immune diseases may result from the variable expression of autoinflammatory and autoimmune factors in disease production, establishing a continuous spectrum of mixed patterns [2]. Connecting molecules or molecular aggregates between them are critical to understanding autoinflammatory-autoimmune disease interactions. Members of the IL-1 family and the inflammasome are key components in crosscommunication between these diseases with mixed components. Mutations in genes related to the inflammasome have been associated with autoinflammation. This multiprotein complex has been associated with organ-specific autoimmunity since a wide spectrum of endogenous danger signals can activate inflammasome products, including IL-1*β*, triggering adaptive immunity pathways [3].

Genetic predisposition involves many loci encoding key immune pathway molecules. These genes are under epigenetic control, influenced by several environmental factors in susceptible individuals. Interleukin-1 seems a critical link between autoinflammatory and autoimmune diseases involving innate and adaptive mechanisms.

Trends for classifying and providing a theoretical framework for all immune diseases that include autoinflammatory and autoimmune nature still hold. However, deeper comprehension of immune-mediated pathologies is required before postulating a unified explicative model.

In this review, we examine current knowledge on the inflammatory role of the IL-1 cytokine family, their association with the inflammasome in autoinflammatory and autoimmune disorder regulation, and the underlying implication of innate and adaptive immunity in diseases with a mixed pathogenic pattern, with particular focus on psoriasis [4].

## 2. The IL-1 Family

Eleven members of the IL-1 family participate in natural immunity and contribute to acute and chronic inflammation. The clinical severity results from the balance between the proinflammatory and anti-inflammatory IL-1 family members in some forms of rheumatic disease [5]. The IL-1*β* is the best-characterized member of the IL-1 family cytokines and potent inflammation mediator in the immune-inflammatory response. The IL-1 family—IL-1*α*; IL-1*β*; IL-18; IL-33; IL-36*α*, *β*, and *γ*; IL-37; and IL-38—includes regulatory factors that modify the intensity of the inflammatory response like decoy receptors, receptor antagonists, and inflammatory pathway signaling inhibitors. The identification of multiple negative regulation pathways of the IL-1/IL-1R family highlighted the need for tight control of the IL-1 family repertoire [6]. The pathophysiological role of IL-1*β* is well established in autoinflammatory diseases, and IL-1*β* and IL-18 are critically associated with severity in various autoimmune and chronic inflammatory pathologies [7].

Inflammasomes comprise a multimolecular complex of specialized intracellular sensors. Mutations in inflammasome-related genes have been associated with autoinflammation and autoimmunity. A broad spectrum of endogenous danger signals can activate inflammasome products, including IL-1*β*, triggering adaptive immunity pathways [3].

The pathophysiological transition results from the imbalance between the proinflammatory activities of IL-1 cytokines and their control mechanisms.

The IL-1 family members (Table 1) play a key role in innate and adaptive immunity and in the pathogenesis of autoimmune and autoinflammatory diseases. Members of the IL-1R-like receptor family include signaling molecules and negative regulators.

## 3. Psoriasis: A Leading Case in Mixed-Pattern Psoriasis Diseases

Psoriasis is considered a systemic chronic inflammatory disease with an immunogenetic basis that can be triggered extrinsically or intrinsically [8, 9]. The disease is characterized by critical interactions between components of the adaptive and the innate immune systems [10, 11].

In recent years, remarkable progress has been made in our understanding of the critical immune pathways involved in psoriasis. Genetic studies have shown that susceptibility to psoriasis involves components of both the adaptive and innate immune systems. Activation of both arms of the immune system is implicated in psoriasis of the skin, while autoimmune adaptive pathogenic immune responses predominate in chronic plaque psoriasis and innate autoinflammatory pathogenic responses dominate in pustular forms of psoriasis, and other clinical subtypes span a spectrum between plaque and pustular psoriasis. This makes psoriasis a mixed autoimmune and autoinflammatory disease, where the balance between the two responses determines the clinical presentation [12].

**Figure 2:**
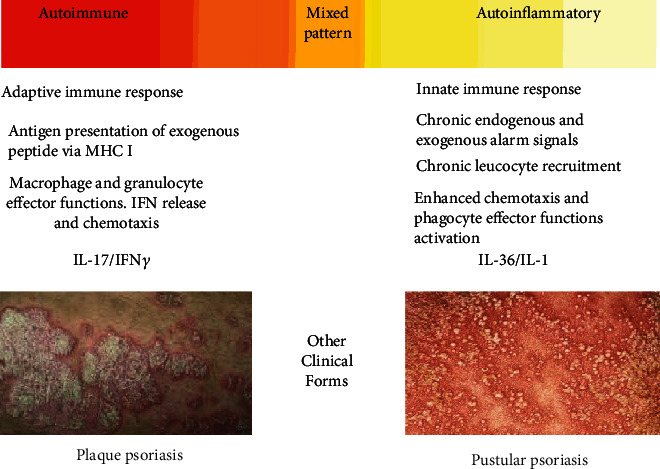
Autoimmune vs. autoinflammatory responses in psoriasis. Complicate interactions between the innate and the adaptive immune systems characterize the pathophysiology of psoriasis. Once settled, the relative contribution of inflammatory and regulatory mediators of adaptive and innate immunity determines the clinical manifestation towards chronic stable vs. highly inflammatory and/or pustular psoriasis. Plaque psoriasis (psoriasis vulgaris) and generalized pustular psoriasis (psoriasis pustulosa) represent autoimmune (IL-17A/IFN-*γ* secretion profile) and autoinflammatory (IL-36/IL-1 secretion profile) response patterns, respectively.

The relative expression of inflammatory mediators, influenced by different IL-1 family members, determines different subclinical patterns along a wide transition spectrum that extends from one type of psoriasis in which the autoimmune component dominates to another in which the autoinflammatory component dominates. Plaque psoriasis presents a typical adaptive immune response, with immune synapsis in secondary lymphoid organs and adaptive leukocyte-effector inflammatory functions in the skin. In contrast, generalized pustular psoriasis is characterized by enhanced chemotaxis-mediated phagocyte infiltration and phagocyte effector functions [13]. The IL-23/IL-17 axis in psoriasis highlighted the strong interaction between cells of the innate immune system (represented by IL-23-producing myeloid antigen-presenting cells) with cells of the adaptive immune system (represented by Th17- and IL-17-expressing cytotoxic CD8+ T cells). The balance between IL-36 and IL-17 partially influences the clinical expression profile (psoriasis vulgaris vs. psoriasis pustulosa) [14] (Figure 2).

### 3.1. Autoimmune Processes in Psoriasis

**Figure 3:**
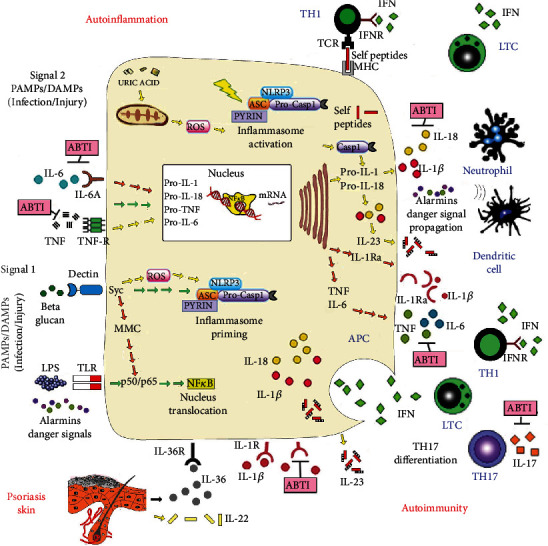
Schematic representation of inflammasome signaling mechanisms in mixed-pattern inflammatory diseases. Inflammatory agents and pathogens trigger the canonical inflammasome pathway. PAMPs and DAMPs are detected by specific innate immune sensors, leading to oligomerization and inflammasome assembly. The therapeutic targets in autoinflammatory diseases are as follows: signal 1 inflammasome activation: surface pattern recognition receptors like Toll-like receptors (TLR) and pathogen-associated molecular patterns stimulate the production of molecules like NF-*κ*B and activate inflammasome assembly through downstream immunologic processes; signal 2 inflammasome activation: crystals in gout, heat-shock proteins, and damaged tissue as in burns, another pathogen- and damage-associated molecular pattern, activate inflammasome assembly through reactive oxygen species (ROS) production and downstream immunologic processes. Certain mediators influence the spectrum of psoriasis, shifting to innate or adaptive immune processes. The interplay between IL-17- and IL-36-driven inflammation seems involved in innate-adaptive immune balance. Inflammasome-induced hyperactive dendritic cells (DC) trigger enhanced T cell responses, preserving antigen and autoantigen presentation and contextualizing T helper cell responses through IL-1*β*, IL-18, and IL-23 secretion. These cytokines trigger Th1/Th17 responses. IL-18 amplifies IFN-*γ* production by Th1 cells and reinforces Th1 differentiation, while IL-1*β* promotes Th17 polarization and IL-17 secretion, causing a mixed autoinflammatory-autoimmune pathology. The image shows the potential sites for antibody-based therapeutic intervention (ABTI). ASC: apoptosis-associated speck protein; ER: endoplasmic reticulum; IFNAR: interferon-associated receptor; IFN: interferon; IL-1: interleukin-1; IL-1 R: IL-1 receptor; IL-1Ra: IL-1 receptor antagonist; IL-6: interleukin 6; IL-6R: IL-6 receptor; IL-18: interleukin-18; JAK: Janus kinase; NLRP3: NOD-like receptor P3; ROS: reactive oxygen species; TLR: Toll-like receptor; TNF: tumor necrosis factor; TNF-R: TNF receptor; ASC: apoptosis-associated speck-like protein containing a CARD; CARD: caspase recruitment domain; DAMP: damage-associated molecular pattern; LPS: lipopolysaccharide; NLR: NOD-like receptor; NOD: nucleotide-binding oligomerization domain; PAMP: pathogen-associated molecular pattern; PYD: Pyrin domain; MMC: multimolecular complex; HMGB1: high-mobility group box 1; TCR: T cell receptor.

Autoimmune signature in psoriasis seems driven by local and systemic Th17 patterns, expressing IL-17A, IL-22, and IFN-*γ* [10] (Figure 3). Chronic-stimulated dendritic cells sustain activation and differentiation of lesional Th17 cells primarily through secretion of IL-23 [15].

Both HLA restriction and T cell peptide specificity are determined by the T cell receptor repertoire. Antigenic stimulation triggers T cells' activation and clonal expansion. In the absence of foreign antigens, clonal T cell expansion likely suggests autoimmunity in inflammatory diseases [16].

Psoriasis seems to be driven by locally prevailing antigens [17]. Environmental factors are mostly rated, including stress, smoking, drugs, and infections [18]. Activated clonal T cells exert disease inflammatory process in combination with locally inflammatory leucocytes. In the last years, putative autoantigens like cathelicidin LL-37, melanocytic ADAMTSL5, lipid antigen PLA2G4D, and keratin 17 have been identified in psoriasis [19–22].

### 3.2. Crosstalk between Adaptive and Innate Immunity in Psoriasis

Complex crosstalk between the innate and adaptive immune systems in psoriasis adds up to antigen-specific exacerbation of inflammation in psoriasis. The accumulating circumstantial evidence suggests that in patients with stable and mild psoriasis, adaptive immunity is likely more prevalent, while innate immunity might contribute more to the active and severe disease, systemic involvement, and comorbid conditions [23] (Figure 3). The coexistence of comorbidities like atherosclerosis in severe psoriasis has been interpreted as a systemic inflammatory reaction to the innate local inflammation in affected tissues [24]. The involved factors are not psoriasis-specific, though they magnify the overall inflammatory burden in patients with severe psoriasis.

The studies addressing the interplay between IL-17- and IL-36-driven inflammation might help understand how certain mediators influence the psoriasis spectrum by shifting innate or adaptive immunity [25]. All IL-36 isoforms (IL-36*α*, *β*, and *γ*) are members of the IL-1 family and are expressed in psoriatic skin [26]. They bind to a specific receptor (IL-1RL2), triggering the transcription of several inflammatory mediators through NF-*κ*B activation.

The IL-36 seems to be associated with the clinical manifestation of specific psoriatic phenotypes. The skin in psoriasis vulgaris differs significantly from that in pustular psoriasis, representing opposite ends of the psoriasis spectrum. The balance between IL-36 and IL-17 might contribute to differential clinical symptoms between the vulgaris and pustulosa forms, in line with the response to given therapies [27].

The IL-23/IL-17 axis is key as it comprises innate immunity (IL-23-producing myeloid cells) and adaptive immunity (Th17- and IL-17-expressing CD8+ T cells). Understanding psoriasis might help shed light on such relationships.

### 3.3. On How IL-1 and IL-8 Participate in Th1 and Th17 Activation by IL-12/23

Some cytokines, including IL-1, induce IL-17 release in human T lymphocytes. Their ability to promote Th17 cells depends not only on the induction of IL-23, IL-6, and TGF-*β* by dendritic cells but also on directly or indirectly activating the inflammasome and inducing IL-1*β*.

IL-12 and IL-23 are extremely important to induce Th1 and Th17, respectively, and their production mediated by antigen-presenting cells is distinctively regulated. Innate immunity-derived stimuli regulate IL-12 and IL-23 production, influencing the induced T lymphocyte phenotype. IL-23 promotes IL-23R expression in myeloid cells and induces proinflammatory TNF-*α* and IL-1*β* cytokine production. Further, IL-23 promotes CD4+ precursors' differentiation to the Th17 effector in the absence of IFN-*γ* and IL-4 [28].

IL-1 promotes lymphocytes' growth and differentiation. The differential expression of IL1R1 in CD4 T lymphocyte subtypes confers different effector functions.

Th17 cells' response to IL-12 and sustained exposure to IL-23 promote Th17 change to the Th1 phenotype [29], indicating the strong environmental influence [30]. Th17-derived Th1 cells are called “nonclassic Th1” and express CD161 and IL1R1 [31]. IL-1*β* and IL-23 combination promotes T cells' production with the Th17 and Th1 phenotype in CD4+ CD161+ and CD4+ CD161- cell fractions. This suggests that Th1 cells respond to IL-1*β* and that CD4+ CD161+ clones in inflamed tissue are able to produce IFN-gamma and express IL1R1 mRNA [32].

IL-8 (CXCL8) participates in the pathophysiology of psoriasis recruiting neutrophils and other inflammatory leukocytes. In fact, IL-8 highly expresses in plaque psoriasis and, up to tenfold, in pustular psoriasis [25].

IL-36, highly expressed in plaque psoriasis, acts on keratinocytes and myeloid dendritic cells [33] and is a potent inducer of the neutrophil CXCL1 and IL-8 chemotactic cytokines. Infiltrating neutrophils play a fundamental role in psoriatic plaque, amplifying the IL-36-mediated autoinflammatory loop in psoriasis [13].

## 4. Association of Inflammasomes with Innate and Adaptive Immunity

Inflammasomes are tripartite complexes comprising a cytoplasmic sensor, an adapter known as ASC, and procaspase-1. Inflammasomes are defined by their cytoplasmic sensor, which includes AIM2, Pyrin, NLRP1, NLRP3, and NLRC4 and belongs to the NOD2-like receptor family. Sensors' diversity and specificity allow inflammasomes to respond to a wide range of either extrinsic (microbial molecules) or intrinsic (danger signals) stimuli.

**Figure 4:**
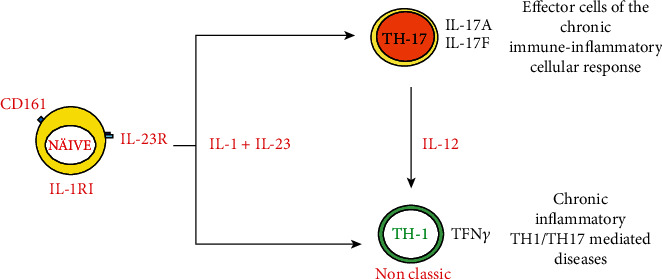
IL-1 and IL23 induce CD4 CD161 precursors' differentiation to classical Th-17 and Th-1 cells in the presence of IL-12. IL-1: interleukin-1; IL-23: interleukin-23; IL-12: interleukin-12; Th: T-helper cells.

The NLRP3 inflammasome is the prototypical and best-characterized inflammasome, and its activation has been sequenced [34]. A first signal, priming, provided by microbial molecules like lipopolysaccharide induces NLRP3 and pro-IL1*β* expression in an NF-*κ*B-dependent fashion. Microbial molecules like toxins or danger signals like monosodium urate offer the second signal and trigger multimerization to make up an inflammasome (Figure 4).

The NLRP3 assembles to ASCs, leading to caspase-1 activation, which induces proteolytic maturation of IL-1*β* and IL-18 and Gasdermin D cleavage. The next pore formation of Gasdermin D in the cell membrane induces pyroptosis, a fast proinflammatory cell death [35]. Pyroptosis associated with the release of IL-1*β*, IL-18, and alarmins contributes to danger signal propagation beyond the damaged or infected cell, recruiting mono- and polymorphonuclear phagocytes (Figure 3). Oligomeric particles may be released from the inflammasome to further amplify the inflammatory response after phagocytosis by surrounding macrophages.

In intact phagocytes, IL-1*β* secretion can occur independently from pyroptosis. Autophagy regulates the inflammasome-processed cytokines, which induce IL-17. Autophagy intersects with the inflammasome-dependent generation of IL-1*β* and IL-18 at different stages. Autophagosomes can remove endogenous inflammasome-activating stimuli, including mitochondrial DNA, ROS, damaged lysosomes, pro-IL-1*β*, and inflammasome components as well. Autophagy inhibits IL-23 secretion due to its effects on IL-1*β* [36].

Involvement in a variety of pathophysiological conditions poses inflammasomes as interesting antibody-based therapeutic intervention targets (Figure 3). From a pathogenetic perspective, they are characterized by chronic activation of the immune system, causing tissue inflammation in genetically predisposed individuals. However, damage-specific effectors are different. In autoinflammatory diseases, the innate immune system directly causes tissue inflammation, while in autoimmune disorders, the innate immune system activates the adaptive immune system, ultimately responsible for the inflammatory process [37].

Some diseases have a mixed autoimmune-autoinflammatory root [38]. Inflammasome dysregulation is associated with autoinflammatory and autoimmune diseases like familial Mediterranean fever, rheumatoid arthritis, psoriasis, and systemic lupus erythematosus [4, 39]. Some immune-inflammatory diseases may reflect a variable expression in the pathogenetic autoinflammatory and autoimmune factors [40].

In an explanatory attempt, Polly Matzinger put forward the danger signal theory. This proposes that the immune system does not so much discriminate between endogenous and exogenous signals but increases responses to danger signals, regardless if they are exogenous pathogenic bacteria or endogenous damaged tissues [41]. However, the hazard model does not adequately explain the exquisite specificity of adaptive immune responses in autoimmune diseases. Recent advances in genetic and molecular studies allow converging to a united classification for all immunological diseases in a theoretical framework. Psoriasis, ankylosing spondylitis, Behcet's syndrome, uveitis, and other diseases show a mixed pattern.

Inflammasome-hyperactivated dendritic cells elicit enhanced T cell responses. They preserve their antigen-presenting function and contextualize T-helper cell responses through IL-1*β* and IL-18 secretion. These cytokines drive Th1/Th17 responses in particular. The IL-18 amplifies IFN-*γ* production by Th1 cells, while IL-1*β* promotes Th17 polarization and IL-17 secretion [42] (Figure 3).

Inflammasome-dependent IL-1*β*-driven Th17 responses are essential for host defense against infections by fungi like Candida albicans. The C-type lectins Dectin-1 are involved in host defense mechanisms against fungal infection, driving inflammatory and adaptive immune responses. Dectin-1 is a type-C lectin receptor that detects *β*-glucans [43]. This leads to Syk-dependent NF-*κ*B activation and NLRP3 inflammasome assembly, while Th17 responses yield immune protection against the pathogen [44]. Notably, Dectin-1 signaling also triggers IL-1*β* production through a noncanonical caspase-8 inflammasome [45].

The divergent roles of IL-1*β* and IL-18 in adaptive immunity setup have drawn much attention to inflammasomes as adjuvants to vaccines. The Th1-mediated humoral responses, cytotoxic T cell/Th1/Th17 immunity, and immune memory can be manipulated using inflammasome-activating ligands [46]. Type I interferons inhibit pro-IL-1 synthesis, promote IL-18 maturation, and, combined with inflammasomes' activation, might aid in modeling protective Th1 responses [47]. The vaccine adjuvant chitosan is a cationic polysaccharide that induces type I IFN production, NLRP3 inflammasome activation, and intense Th1 responses. More studies are needed to better understand the role of inflammasomes in pathological and protective immunity.

Apoptosis and pyroptosis are two well-studied cell death patterns, traditionally believed as unrelated. Emerging evidence shows their extensive interrelation as converging pathways, activating the same cell death effector, the pore-forming protein Gasdermin D [48].

Pyroptosis is the inflammatory cell death triggered by intracellular detection of signs of damage or pathogens [49]. Pyroptotic cells show swelling, fragmented genetic material, membrane pore formation, plasma membrane rupture, and release of inflammatory mediators and cytoplasmic content to the extracellular space [50]. Lipopolysaccharide, a hallmark of the gram-negative bacterial cell wall, is a prototypical trigger of immune cell pyroptosis. Pyroptosis starts with the innate TLR4 activation step. This induces NF-*κ*B activation and translocation to the nucleus to boost gene transcription for precursors pro-IL-1*β*, pro-IL-18, and procaspases and intracellular Nod-like receptors' transcription. In psoriatic lesions, Dectin-1 upregulation seems under the control of psoriasis-associated cytokines, while its role in the biology of skin inflammation and infection is to be explored [51].

The second signal induces oligomerization of intracellular complexes called inflammasomes [52], which facilitate pro-IL-1*β* and procaspase-1 maturation into their active forms. While IL-1*β* is released and induces a proinflammatory state, caspase-1 breaks down the cytoplasmic gasdermin D, forming pores in the membrane and triggering cell death through cytoplasmic components' leakage.

Autophagy is a self-degrading process required to restore cell homeostasis when menacing factors are detected [53]. This ubiquitous lysosomal degradation mechanism removes damaged proteins and organelles, contributes to antigen presentation to the cell surface, protects against genome instability, and prevents tissue damage. Autophagy is of physiological relevance, helping defend against damaging stress while leading to pathology when in excess or defect [54].

As an essential homeostatic mechanism, autophagy is upregulated in response to environmental and pharmacological triggers. It has a very important role in cancer, neurodegeneration, diabetes, and liver and autoimmune diseases. Molecular elements that lead to this type of cell death also collaborate in the stress response.

In the immune system, autophagy serves as a source of peptides for antigen presentation [55], provides a mechanism for the absorption and degradation of intracellular pathogens, and is a key regulator of inflammatory cytokines. It is also involved in regulating inflammasome activation and helping remove inflammasome components and endogenous activators [56] and plays a role in determining IL-1*β* fate in autophagosomes. Present understanding suggests that autophagy is a critical regulator of inflammasome activation and IL-1 family cytokines' release [57].

## 5. Inflammasome-Induced IL-1 Promotes IL-17-Mediated Responses

An inflammasome is a multiprotein complex that contributes to defense against pathogens and repair during inflammatory processes while producing inflammatory diseases under aberrant chronic conditions. Inflammasome assembly triggers caspases' activation, setting off inflammatory cytokines, including IL-1 activation.

The finding of IL-17 and IL-17-secreting T cells has improved our understanding of the T cell role in autoimmune and other inflammatory diseases. The Th1 cells were first considered key pathogenic T cells in many autoimmune diseases. However, mice deficient in IFN-*γ* or IL-12 signaling had exacerbated symptoms in certain autoimmune diseases [58].

Dendritic cells associated with inflammasome hyperactivity boost T lymphocyte activity (Th1/Th17) through increased IL-1*β*, IL-18, and IL-23 release. IL-18 amplifies Th1 cells' IFN-*γ* production and enhances Th1 differentiation while IL-1*β* promotes Th17 polarization and IL-17 release, triggering a pathological autoinflammatory and autoimmune profile.

Besides, IL-1 and IL-23 (Figure 4) can induce and activate human Th1/Th17 cell differentiation. IL-1 can induce cells of the innate immune system to produce IL-6, which stimulates naïve T cell differentiation to Th17 [59].

## 6. Conclusions

From a pathogenic perspective, most autoinflammatory and autoimmune diseases share a chronic aberrant immune system activation, which leads to tissue inflammation and damage of varying magnitude in genetically predisposed individuals. IL-1 has grown into a complex, multifaceted family of cytokines with complex regulatory mechanisms and diverse functions in health and disease.

IL-1 and inflammasome are strongly associated with adaptive and autoimmune disorders. The role of the inflammasome-associated IL-1 cytokines' family in shaping adaptive immune responses is now well-established regarding the differentiation of Th17 cells and promoting effector functions of Th1 cells and CD8 T cells. In addition, cell lysis triggers inflammasome activation, releasing additional DAMPs and self-antigens, linking autoinflammation and autoimmunity. The contribution of IL-1 and associated molecules to inflammasome regulation needs exploration to improve our understanding of inflammatory diseases.

The relevance of the IL1-related cytokines has outreached classic immunopathology and is a critical bridge to understanding mixed-pattern diseases.

Novel therapeutic intervention strategies may be anticipated after deepening our understanding of inflammatory disorders and the molecular pathways of autoinflammation, autoimmunity, and immune homeostasis regulation.

## Figures and Tables

**Table 1:** IL-1 family of ligands and receptors.

IL-1 family	Specific receptor	Coreceptor	Function
IL-1*α*, IL-1*β*	IL-1R1	IL-1RAP	Proinflammatory
IL-1*β*	IL-1R2	IL-1RAP	Anti-inflammatory
IL-1Ra	IL-1R1	Not available	Anti-inflammatory
IL-18	IL-18R1	IL-18RAP	Proinflammatory
IL-33	IL-1RL1	IL-1RAP	Proinflammatory
IL-36*α*, *β*, *γ*	IL-1RL2	IL-1RAP	Proinflammatory
IL-36Ra	IL-1RL2	IL-1RAP	Anti-inflammatory
IL-37	IL-18R1	SIGIRR	Anti-inflammatory
IL-38	IL-1RL2	IL-1RAPL2	Anti-inflammatory

## Data Availability

The data supporting this review are from previously reported studies, which have been cited.

## Abbreviations

HLAs: Human leukocyte antigens
IL-: Interleukin
Th17: T helper lymphocyte
CD8+: Cytotoxic T cells
IFN-*γ*: Interferon gamma
NF-*κ*B: Nuclear factor kappa-light-chain-enhancer of activated B cells
ASC: Apoptosis-associated protein
AIM: Absent in melanoma
NLRP: NLR family pyrin domain containing
NOD: Binding oligomerization domain
ROS: Reactive oxygen species
TLR4: Toll like-4 immune receptor.
